# Bioassay-Guided Isolation of 2-[p-(2-Carboxyhydrazino)phenoxy]-6-(hydroxymethyl)tetrahydro-2H-pyran-3,4,5-triol from *Oroxylum indicum* and the Investigation of Its Molecular Mechanism Action of Apoptosis Induction

**DOI:** 10.3390/ph15050559

**Published:** 2022-04-30

**Authors:** Asem Robinson Singh, Salam Asbin Singh, Thangjam Davis Singh, Naorem Tarundas Singh, Takhellambam Chanu Machathoibi, Okram Mukherjee Singh, Lisam Shanjukumar Singh

**Affiliations:** 1 Cancer and Molecular Biology Division, Department of Biotechnology, Manipur University, Canchipur, Imphal 795003, Manipur, India; asem.robinson@gmail.com (A.R.S.); salam.asbin@gmail.com (S.A.S.); davis.thangjam8@gmail.com (T.D.S.); tarundasnaorem@gmail.com (N.T.S.); machathoibi2008@gmail.com (T.C.M.); 2 Department of Chemistry, Manipur University, Canchipur, Imphal 795003, Manipur, India; omsingh@manipuruniv.ac.in

**Keywords:** *Oroxylum indicum*, oroxyquinone, traditional medicine, bioassay-guided fractionation, apoptosis, caspase 3-independent, anti-metastatic

## Abstract

The leaf crude extract of *Oroxylum indicum* (L.) Kurz induces genomic DNA fragmentation, comet formation, and the inhibition of cell proliferation in the prostate cancer cell line PC3, as assessed by agarose gel electrophoresis, comet assay and MTT assay, respectively. The bioactive compound was purified through bioassay-guided fractionation using preparative HPLC and MTT assay. The light brown and water-soluble compound was characterized using 1H and 13C nuclear magnetic resonance (NMR), Fourier transform infrared (FT-IR), and electrospray ionization (ESI) mass spectrometry. The compound was identified as a glycosylated hydroquinone derivative, 2-[p-(2-Carboxyhydrazino)phenoxy]-6-(hydroxymethyl) tetrahy-dro-2H-pyran-3,4,5-triol (molecular formula, C13H18N2O8; molecular mass = 330). The identified phytocompound has not been reported earlier elsewhere. Therefore, the common name of the novel anticancer phytocompound isolated from *Oroxylum indicum* in this current study is oroxyquinone. The half-maximal inhibitory concentration (IC_50_) of oroxyquinone on PC3 cells was 58.9 µM (95% CI = 54.5 to 63.7 µM). Treatment of PC3 cells with oroxyquinone induced genomic DNA fragmentation and chromatin condensation, increased in the annexin-V positive cells, arrested the cell cycle at S phases, and inhibited the cell migration; as assessed by comet assay, DAPI staining, flow cytometry and a wound healing assay, respectively. On the investigation of the molecular mechanism of the induction of apoptosis, the results indicated that oroxyquinone induced caspase-3 and PARP independent apoptosis but through the p38 pathway and the localization of AIF into the nucleus. The present study identifies a novel anticancer molecule and provides scientific evidence supporting the therapeutic potency of *Oroxylum indicum* for ethnomedicinal uses.

## 1. Introduction

Cancer ranks as one of the leading causes of mortality and morbidity worldwide, with most cases being seen in developed nations. It has a mortality rate of one in eight deaths worldwide, which is a figure that surpasses the cumulative death rate of AIDS, tuberculosis, and malaria [1]. According to the World Health Organization (WHO), in 2018, 18.1 million people worldwide had cancer, and 9.6 million died from the disease. By 2040, those figures will nearly double, with the greatest increase in low-income countries, where more than two-thirds of the world’s cancers will occur. Cancer was the cause of about 30% of all premature deaths from non-communicable diseases among adults aged 30–69. The total number of cases diagnosed in India between 2017 and 2018 was 784,821 (an increase of 324%).

With an expected rise of more than 70% of new cases in the coming two decades, finding a solution to the medical problem is a concern for healthcare organizations around the globe. In cancer prevention and treatment, traditional medicines have played a significant role for centuries. A few of the plant-derived anticancer agents that are in use today for cancer management are alkaloids derived from *Vinca rosea*, taxanes from *Taxus brevifolia*, camptothecin-derived drugs from *Camptotheca acuminata*, and epipodophyllotoxins of *Podophyllum peltatum*. However, a vast potential lies ahead for the discovery of new phytochemicals that can be used as chemotherapeutic drugs.

Various phytocompounds were characterized to have different molecular mechanisms of inducing apoptosis in a caspase-dependent or -independent manner, including the generation of reactive oxygen species (ROS) or through the mediation of Endonuclease G (Endo-G) or Apoptosis-Inducing Factor (AIF). Galluzzi et al., 2012, published a detailed description of cell death mechanisms in which various molecular pathways were discussed [2]. As cancer cells have a means of evading natural cell death, finding an alternate pathway to induce cell mortality remains an open avenue for drug discovery. Our present study envisages the determination of the molecular pathway(s) involved in apoptosis induced by a leaf extract of *Oroxylum indicum* (OI).

*Oroxylum indicum* (L.) Kurz (Kingdom-Plantae, Class- Magnoliophyta, Order- Lamiales, Family- Bignoniaceae, genus- Oroxylum, species-Indicum, local name shamba) is found throughout the Asian sub-continent, including India. Different parts of *Oroxylum indicum* are found to be used extensively in different forms of Indian traditional medicine, such as Ayurveda and Unani, and Traditional Chinese Medicine (TCM) for the prevention and treatment of several diseases, such as jaundice, arthritic and rheumatic problems, gastric ulcers, tumours, respiratory diseases, diabetes, diarrhoea and dysentery, among others. Stem bark, root bark, seeds, and leaves are the most commonly used parts to treat various ailments like rheumatoid arthritis, gout, wounds, and gastric ulcers [3]. In the recent past, several studies were undertaken on the properties and pharmacological activities of OI’s phytochemicals, and suggested that OI’s phytochemicals have anticancer, free-radical scavenging, and immune-stimulant effects. Costa Lotufo et al., 2005, reported the cytotoxic activity of stem bark extract of OI on murine melanoma cells (B-16) and Human leukemia cells (HL-60) [4] as well as in cervical cancer cells (HeLa) by Moirangthem et al., 2013 [5]. The antimutagenic property of the plant extract has also been described by Nakahara et al. [6]. Naveen Kumar et al. described the anticancer activity of stem bark extract of the plant in breast cancer cell line MDA-MB-231, with petroleum extract exhibiting the highest activity [7]. More recently, the focus on the molecular mechanism of anticancer activity of the plant parts of OI revealed the involvement of P13K/Akt/PTEN pathways [8], and the inhibition of NF-κB, COX-2, RASSF7 and NRF2 [9] in HeLa cells.

In an attempt to isolate all of the present flavonoids in the seeds of OI, Kruger and Ganzera isolated six major flavonoids, including baicalein, chrysin, oroxylin, and their derivatives recently [10]. The baicalein-rich fraction from OI was shown to have anticancer activity in HPV-infected cervical cancer cells [11]. Furthermore, the compound was shown to induce mitochondrial-mediated apoptosis through the MAPK pathway [12]. Leaf and seed extracts of OI have been seen to have an anti-metastatic effect on breast cancer cells through the modulation of the Rac1 pathway [13]. Most of the studies on the anticancer activity of OI are based on the stem, root, bark or seeds of the plant, whereas the leaves remain largely unexplored. The present study focuses on finding a novel anticancer phytocompound of OI on PC3 cells.

## 2. Results

### 2.1. Leaf Crude Extract of OI Inhibits the Proliferation of PC3 Cells

**Figure 1:**
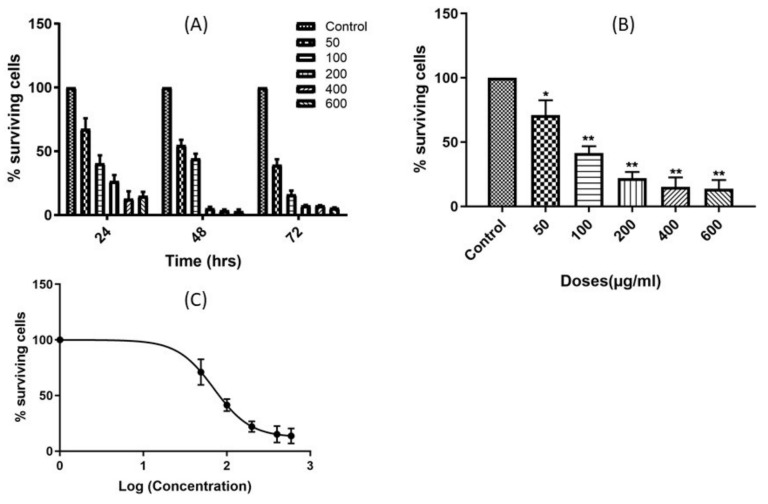
Leaf crude extract of OI inhibits proliferation on PC3: (**A**) A leaf crude extract of OI was treated to PC3 at different doses (50-, 100-, 200-, 400- and 600 µg/mL) for 24-, 48- and 72 h, and its effect on cell proliferation was assessed by MTT assays. (**B**) The half-maximal inhibitory concentration (IC_50_) of leaf crude extract for the inhibition of cell proliferation was measured on PC3 cells by treating the cells with the doses, as above, for 24 h, and MTT assays were conducted. The control (Cont) cells were treated with PBS under identical conditions to the treated cells. (**C**) IC_50_ was measured using GraphPad Prism 8. The percentage of viable cells of each treated group was calculated after taking the viable numbers of control cells to be 100%. The p-values were calculated using Student’s “*t*” test. The bars indicate SD, * indicates a *p*-value > 0.05 and ** indicates a *p*-value > 0.01.

PC3 cells were treated with different doses of extract for different time points. The results showed the significant inhibition of cell proliferation in a dose- and time-dependent manners (Figure 1A). Therefore, the results indicate that the leaf extract of OI has a cytotoxic activity towards PC3 cells. Furthermore, the IC_50_ of the leaf extract was determined by treating the cells with different doses of the extract for 24 h, and the cell viability was determined by an MTT assay, as above. The result also showed that the IC_50_ value of the leaf crude extract of OI was 69.99 µg/mL (95% CI = 57.11 to 85.77) (Figure 1B,C).

### 2.2. The Leaf Crude Extract of OI Induces Apoptosis in PC3 Cells

**Figure 2:**
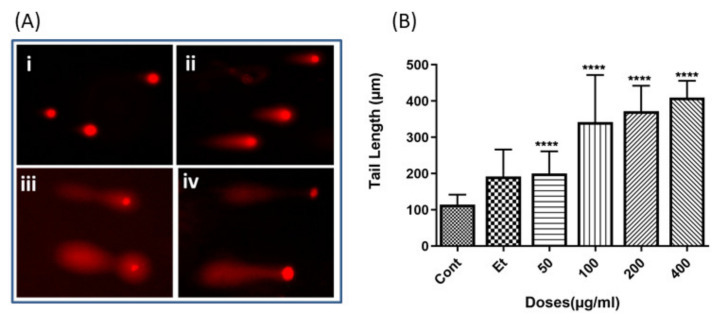
Leaf crude extract of OI triggers gDNA fragmentation in PC3 cells: PC3 cells were treated with 50, 100, 200 or 400 µg/mL of leaf crude extract dissolved in PBS. The negative control cells (Cont) were treated with PBS. After treatment (**A**), comet assays were conducted as described in the Materials and Methods: (i) control, (ii) 50 µg/mL, (iii) 200 µg/mL, (iv) and 400 µg/mL. (**B**) The tail length of 25 comets in each group was measured, and the mean and standard deviation were calculated. *p*-values were calculated for each group, comparing with the control group using Student’s “*t*” test. **** indicates *p*-values > 0.0001. The bars indicate SD.

In order to further investigate the mechanism of inhibition of cell proliferation induced by the leaf crude extract of OI, genomic DNA (gDNA) fragmentation (DNA ladder formation)—which is a hallmark of cell apoptosis—was analyzed in PC3 cells, as described in the Materials and Methods. The results showed that the leaf crude extract induced gDNA fragmentation in PC3 cells at 24 h of treatment in a dose-dependent manner (Supplementary Figure S1). The finding was further confirmed by a comet assay, which is based on single-cell gDNA fragmentation, as described in Section 4 (Figure 2A). The results indicated that in PC3 cells, even at a low dose (50 µg/mL), gDNA fragmentation was observed, and the length of the comet tails increased with the dose of the leaf crude extract (Figure 2B). The result of the inhibition of cell proliferation is in accordance with the results of the gDNA fragmentation. Therefore, the results clearly indicated that the crude leaf extract of OI inhibits cell proliferation through the induction of programmed cell death, apoptosis.

### 2.3. Bioassay-Guided Fractionation Purified a Novel Bioactive Compound

**Figure 3:**
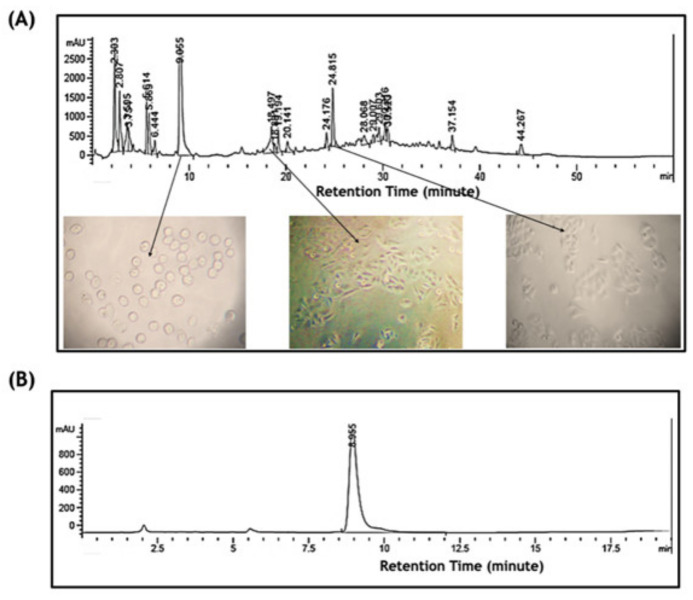

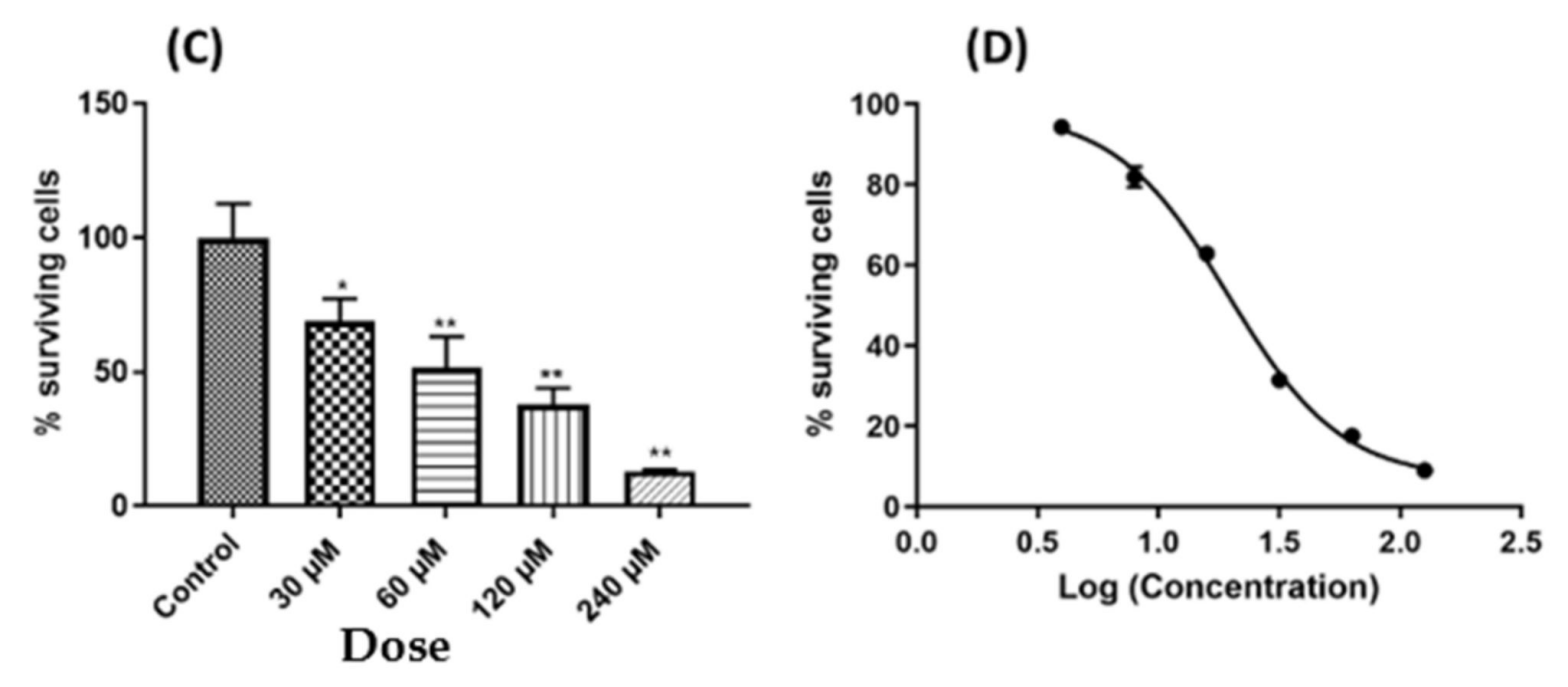
Purification of the bioactive compound using HPLC. The leaf extract was subjected to silica gel chromatographic fractionation, and then each fraction was subjected to a bioassay, as described in Section 4. (**A**) Fraction 3 of the silica gel chromatography was further subjected to preparative HPLC fractionation, and single peaks were further subjected to a bioassay. (**B**) A single peak of a 9.052 min retention time showed anticancer activity, and it was further analyzed on HPLC for purity. (**C**) The purified phytocompound collected as a single peak on HPLC was subjected to an MTT assay after treating the PC3 cells with 30, 60, 120 and 240 µM for 24 h. The percentage of viable cells of each treated group was calculated after taking the viable numbers of the control cells as 100%. The *p*-values were calculated using Student’s “*t*” test. The bars indicate SD, * indicates a *p*-value > 0.05, ** indicates *p*-value > 0.01. (**D**) The IC50 for the purified phytocompound was measured using GraphPad Prism 8 software.

In order to purify the bioactive compound of the leaf of OI that induces cell apoptosis, a Bioassay Guided Fractionation (BGF) employing silica gel chromatography, preparative HPLC, and a high-throughput MTT assay was used as described in the Materials and Methods. Twelve fractions of 50 mL each collected from silica gel chromatography were assessed for the inhibition of cell proliferation by MTT assays. The third and fourth fractions out of 12 obtained from silica gel chromatography showed the inhibition of the cell proliferation of PC3, whereas other fractions showed no activity (data not shown). Both fractions from silica gel chromatography were further analyzed and fractionated on preparative HPLC using a C-18 column, as described in the Materials and Methods. All of the major single peaks of the HPLC profiles were collected in a 200 µL volume using a fraction collector, and were assessed for the inhibition of cell proliferation by MTT assays, as above (data not shown). The single peak of a 9.052 min retention time obtained from the third fraction possessed the inhibition of cell proliferation activity, as assessed by MTT assay, and induced cell shrinking, as assessed by morphological changes under a light microscope (Figure 3A). We further analyzed the purity of the compound on HPLC. The analyses showed a clear and sharp single peak, indicating a single compound (Figure 3B).

The IC_50_ of the purified phytocompound for the inhibition of cell proliferation was determined on PC3 cells by MTT assays. The results revealed that the purified phytocompound has an IC_50_ value of 58.9 µM (95% CI = 54.5 to 63.7 µM) (Figure 3 C,D).

### 2.4. Chemical Characterization

**Figure 4:**
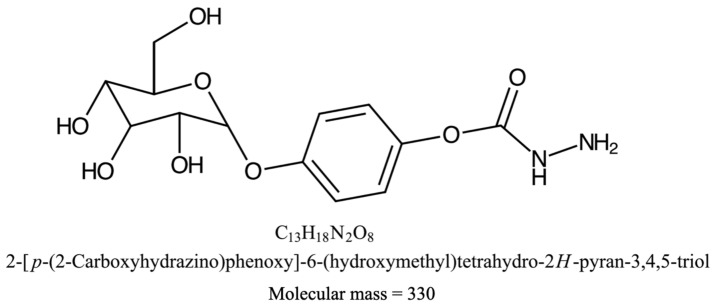
Chemical structure. After the NMR, HRMS, and FT-IR spectra were analyzed, the chemical structure and IUPAC name of the purified phytocompound were generated using ChemDoodle Software (iChemLabs, Ver. 9.0.3).

Light Brown solid; 1H NMR (500 MHz, CDCl3, δ ppm): 7.37–7.42 (m, 1H), 8.32 (s, 3H), 6.85 (d, J = 8.3 Hz, 2H), 5.99 (d, J = 8.3 Hz, 2H), 4.19 (d, J = 10.3 Hz, 1H), 3.96 (m, 1H), 3.84 (m, 1H), 3.59 (m, 1H), 2.74 (m, 1H), 2.65 (m, 1H), 2.56 (m, 1H), 2.21 (m, 4OH); 13C NMR (125.8 MHz, CDCl3, δ ppm): 35.7, 39.3, 42.3, 59.5, 66.2, 69.8, 74.8, 80.8, 127.7, 150.1, 139.1, 153.3, 171.1, 200.9; IR (KBr) (ν max, cm^−1^): 1099.4, 1382.9, 1589.3, 3336.8 cm^−1^; MS: *m*/*z* = 330 (M+). Anal. Calcd for C13H18N2O8; C, 72.16; H, 3.78; S, 12.04. Found: C, 47.27; H, 5.49; N, 8.48; O, 38.75 (Supplementary Figures S2 and S3). On the basis of these observations, the purified compound is proposed to be 2-[p-(2-Carboxyhydrazino)phenoxy]-6-(hydroxymethyl) tetrahy-dro-2H-pyran-3,4,5-triol. The chemical structure elucidated by ChemDoodle Software (iChemLabs, Ver. 9.0.3) is shown in Figure 4. The analysis of 2-[p-(2-Carboxyhydrazino)phenoxy]-6-(hydroxymethyl) tetrahy-dro-2H-pyran-3,4,5-triol using chemical databases—namely SciFinder, ChemSpider, Pubchem and Pubmed—revealed that the purified phytocompound is not found in the these chemical databases, indicating that it is a novel compound. Therefore, the common name of this novel phytochemical is given as Oroxyquinone for convenience.

### 2.5. Oroxyquinone Induces Apoptosis in PC3 Cells

**Figure 5:**
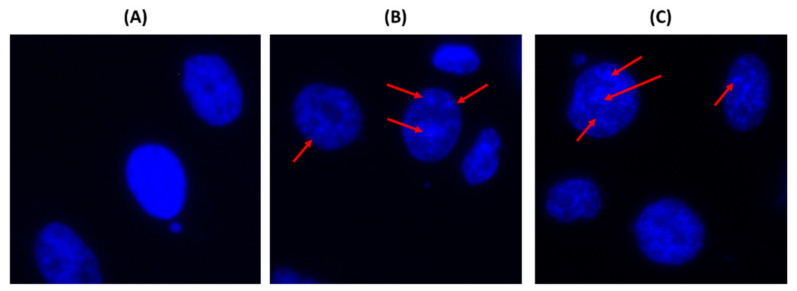
Oroxyquinone-induced chromatin nucleation: PC3 cells treated with PBS (**A**), 30 µM (**B**) and 60 µM (**C**) of oroxyquinone were stained with DAPI and observed under a fluorescent microscope. Nucleation, indicated by red arrows, was observed in cells treated with the purified compound.

**Figure 6:**
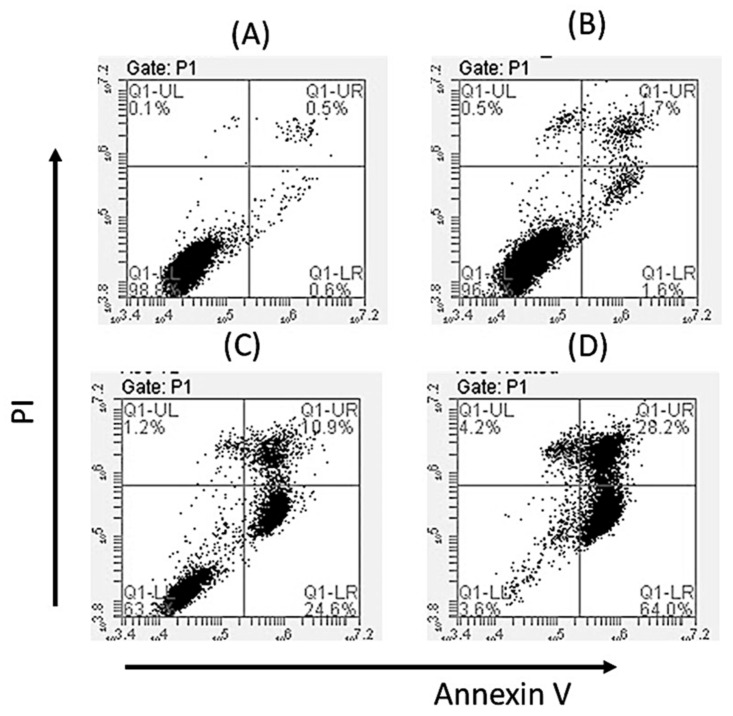
Oroxyquinone sensitizes PC3 cells to Annexin V: PC3 cells treated with PBS (**A**), or 30 µM (**B**), 60 µM (**C**) and 120 µM (**D**) oroxyquinone were stained with Annexin V (FITC) and PI to determine apoptosis. An increase in the number of annexin V-positive cells was observed from 24.6% to 64.0% of the total cells with an increased dose of oroxyquinone.

In order to determine if the cytotoxicity activity of Oroxyquinone is due to the induction of apoptosis, we analyzed chromatin condensation by DAPI staining in PC3 cells after treatment with 30 and 60 µM of the compound under a fluorescent microscope. We observed an increase in the nucleation of chromosomes with the increase in the dose of oroxyquinone (Figure 5). In order to confirm the effect of oroxyquinone on gDNA fragmentation at the cellular level, a comet assay was perfomed. The result showed that the purified oroxyquinone induces gDNA fragmentation in a dose-dependent manner (data not shown). Furthermore, cell apoptosis was analyzed by flow cytometry after staining with PI and annexin-V conjugated with FITC. The results showed a statistically significant increase in the number of annexin-V positive cells in a dose-dependent manner on oroxyquinone (Figure 6).

### 2.6. Oroxyquinone Induces Cell Cycle Arrest in PC3 Cells

**Figure 7:**
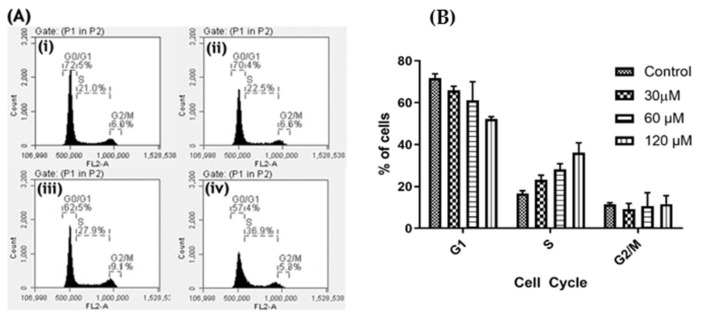
Oroxyquinone triggers cell cycle arrest: PC3 cells were cultured and treated with different doses of OI, as indicated, or PBS (Control) for 24 h. (**A**) The cells were subjected to propidium iodide staining to measure the DNA content in each cell by flow cytometry. (**B**) The percentage of cells in each phase of the cell cycle was compared between the control and treated cells. The number of cells in the S phase increased with an increase in the dose in a statistically significant manner (*p* < 0.05).

In order to further investigate the effect of oroxyquinone on cell division, we conducted a cell cycle analysis in PC3 on a flow cytometer, as described in the Materials and Methods. The results showed that oroxyquinone induced cell cycle arrest at the S phase in a dose-dependent manner in PC3 cells. The numbers of PC3 cells at the S phase were 16.6 ± 1.46%, 23.27 ± 2.27%, 28.23 ± 2.65% and 36.1 ± 4.75% at 0, 30, 60 and 120 µM of oroxyquinone treatment, respectively (Figure 7A,B). The number of cells at the G0/G1 phases decreases with doses of oroxyquinone. However, the number of cells remains almost unchanged at the G2/M phases. Therefore, the findings clearly indicate that oroxyquinone triggers cell cycle arrest at the S phase, which may possibly be due to DNA fragmentation induced by the novel phytocompound.

### 2.7. Oroxyquinone Inhibits PC3 Cell Migration

**Figure 8:**
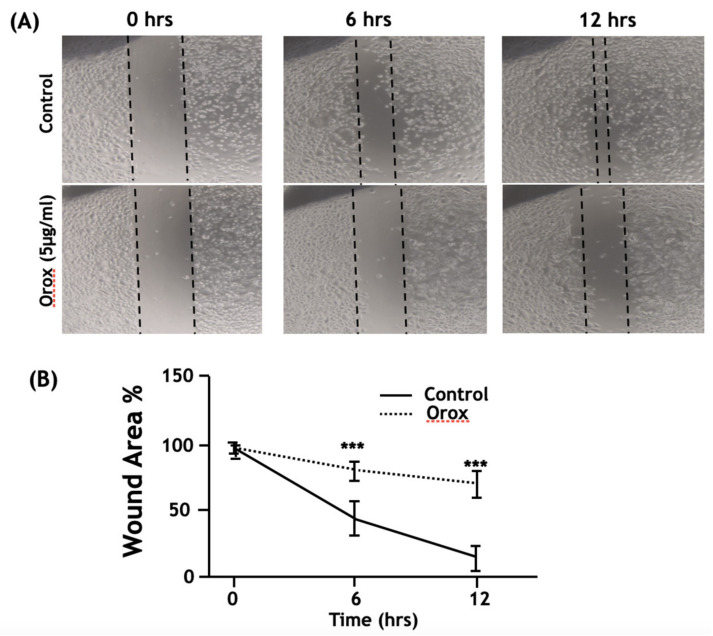
Oroxyquinone inhibits PC3 cell migration. The PC3 cells were cultured, and a wound healing assay was conducted as described in the Materials and Methods by treatment with 5µg/mL of oroxyquinone (Orox) or PBS (Control). (**A**) The pictures represent the images of the same cells of each group recorded at 0, 6, and 12 h of treatment. The dark marks on the upper left side of the image were to make sure the images were recorded on the same field each time. (**B**) In order to measure the area of the wound, the length of the scratches was measured on the light microscope and plotted on a graph. The wound area of the control at 0 hr was taken as 100%, and means and SD were calculated. *p*-values were calculated comparing with the control values of the same h of treatment by students’ “*t*” test. *** indicates *p*-value > 0.001.

In order to investigate the anti-metastatic potential of oroxyquinone, a wound healing assay was conducted on PC3, which is highly metastatic. Initially, MTT assays were performed on PC3 cells with low doses of oroxyquinone (data not shown), and 5 µM, which does not exhibit the inhibition of cell proliferation, was selected for the wound healing assay. The results showed that oroxyquinone (5 µM) significantly reduced the PC3 cell migration compared to the control (untreated) cells by approximately 35% at 6 h and approximately 55% at 12 h (Figure 8A,B). The findings strongly suggested that the novel phytochemical purified from the leaf of OI has anti-metastatic potency toward highly metastatic cells in addition to its antiapoptotic potency.

### 2.8. Oroxyquinone Induces Cells Apoptosis in PC3 Cells in a Caspase-3-Independent Mechanism

The activation of caspase-3 and the deactivation of PARP upon treatment were assessed using Western blotting to determine the molecular mechanisms of the apoptosis induced by oroxyquinone in PC3 cells. The cleavage of caspase-3 was not observed in PC3 cells even at the highest dose used (120 µM). The further cleavage of PARP, which is the downstream protein of caspase-3, was investigated. As expected, the cleavage of PARP was not observed in PC3 cells at any dose used (Supplementary Figure S4). Therefore, the results clearly suggested that oroxyquinone induces apoptosis in a caspase-3 and PARP-independent manner.

**Figure 9:**
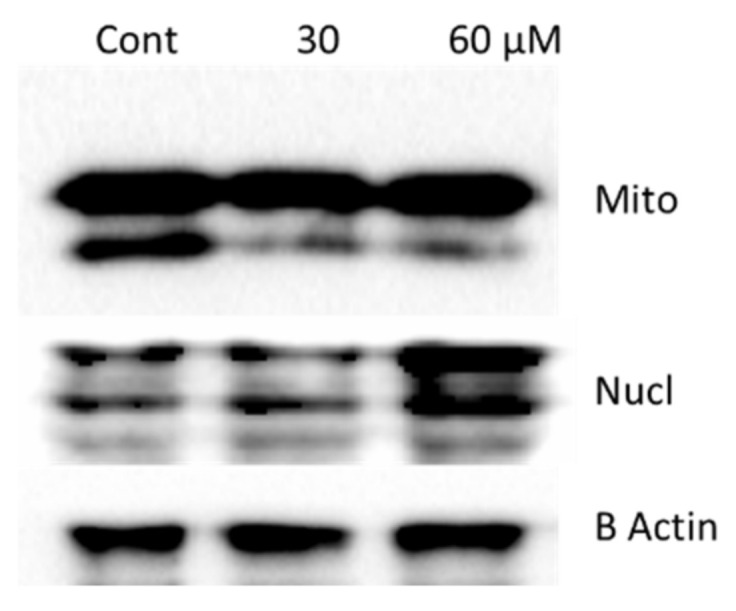
Oroxyquinone caused the nuclear localization of AIF: PC3 cells were treated with PBS (Cont), 30 and 60 µM oroxyquinone, and incubated for 24 h. The cells were lysed, and the cellular fractions were separated. The AIF in the mitochondrial (Mito) and nuclear (Nucl)-enriched fractions was detected by Western blotting. An increase in AIF localization to the nucleus was observed in 60-µM treated cells compared to the control. A subsequent decrease in the AIF concentration in the mitochondria was also observed accordingly.

Because the above results suggested that oroxyquinone induces apoptosis in PC3 cells without the involvement of caspase-3 and PARP proteins, we further investigated the involvement of Apoptosis Inducing Factor (AIF), as it is one of the probable proteins that causes DNA fragmentation when localized from mitochondria to the nucleus during apoptosis. We analysed the AIF localization (mitochondrial and nuclear localization) after treatment of different doses of oroxyquinone (30 µM and 60 µM); the mitochondrial- and nuclear-enriched proteins were fractionated, and AIF localizations were analyzed by Western blot against anti-AIF antibodies. The results indicated an increase in AIF in the nuclear fraction, along with a decrease in the mitochondrial fraction in a dose-dependent manner (Figure 9).

### 2.9. Oroxyquinone Activates MAPK Proteins

**Figure 10:**
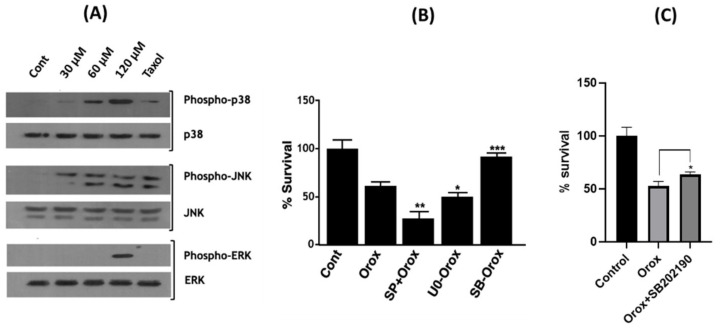
Oroxyquinone induces apoptosis via MARKs pathways. (**A**) PC3 cells were cultured and treated with different doses of oroxyquinone; the total proteins were extracted and separated by SDS-PAGE, and were immunoblotted using antibodies against total and phosphorylated forms of p38, JNK, and ERK. (**B**) PC3 cells were cultured and treated with the specific inhibitors SP600125 (SP), U0126 (U0), and SB203580 (SB) for JNK, ERK, and p38, respectively, for 24 h, and MTT assays were performed. (**C**) The involvement of p38 in apoptosis in PC3 cells treated with oroxyquinone was revalidated using p38 inhibitor SB202190. The *p*-value was calculated by comparison to the oroxyquinone-treated cells using Student’s “*t*” test. The bars indicate SD, * indicates a *p*-value > 0.05, ** indicates a *p*-value > 0.01, and *** indicates a *p*-value > 0.001.

Furthermore, the signalling pathways followed by oroxyquinone for cell apoptosis induction were investigated by Western blotting using antibodies against the total and phosphorylated ERK, JNK, and p38 proteins. The results showed that in PC3 cells, oroxyquinone activates p38 and JNK proteins at all of the doses used (30, 60, and 120 µM) in a dose-dependent manner (Figure 10A, upper and middle panels). However, ERK is not activated at lower doses (30 and 60 µM) of oroxyquinone, but only at the highest dose (120 µM) used (Figure 10A, lower panel). To confirm the functional roles of p38, JNK, and ERK involved in the oroxyquinone-induced apoptosis in PC3 cells, MTT assays were conducted in the presence and absence of specific inhibitors of p38, JNK, and ERK. The results revealed that the presence of a specific inhibitor of p38 (SB203580) abrogates the oroxyquinone-induced inhibition of PC3 cell proliferation. In contrast, the presence of specific inhibitors for JNK (SP600125) or ERK (U0126) enhances the inhibition of PC3 cell proliferation (Figure 10B). This result was further confirmed by using another p38-specific inhibitor (SB202190) which yielded a similar result, confirming the role of p38 in apoptosis induction by oroxyquinone (Figure 10C).

## 3. Discussion

OI has been used in many folk medicines in India [14,15], Bangladesh [4], Vietnam [16] China [3] and Thailand [17] for the treatment of cancer, gastric ulcer, fever, arthritis, etc. It has also been shown to possess several other medicinal properties [18,19,20,21,22,23,24,25]. More recently, a study on the anticancer activity of the plant has gained interest, as evidenced by the number of reports. An attempt to purify the bioactive phytocompound with anticancer activities on breast cancer cells was made by Rajkumar and colleagues. However, the molecular mechanism of cell death associated with the compound was not studied [26]. Bicalein, isolated from the fruits of OI, has been shown to induce HL-60 cell apoptosis, as assessed by a Tunnel assay and a DNA fragmentation assay. However, the molecular mechanism was not elucidated. The anticancer activity of the crude extract of the plant is further supported by many other studies [5,7,19,27]. Moreover, most of the studies are based on extracts of the root, stem, bark or seed of OI.

The current study has shown that the leaf crude extract of OI has potent antiproliferative activity in PC3 cells in a time and dose-dependent manner, with an IC_50_ value of 69.99 µg/mL (95% CI = 57.11 to 86.77), as shown in Figure 1. The fragmentation of gDNA was assessed by an agarose gel DNA fragmentation (DNA ladder formation) assay, as well as single cell-based comet assays in PC3 to investigate if the inhibition of the cell proliferation attributes to apoptosis. The gDNA fragmentation is one of the hallmarks of cells undergoing apoptosis. The dose-dependent gDNA fragmentation induced by the crude leaf extract observed in both the gel DNA fragmentation and comet assays suggests that the extract induces cell apoptosis (Figure 2 and Figure S1). Therefore, we developed Bioassay Guided Fractionations (BGF), employing silica gel and the powerful preparative HPLC fractionation, followed by a high-throughput MTT assay to purify and characterize the bioactive phytocompound from the leaf of OI that induces cells apoptosis (Figure 3A). The method of BGF has been put into practice for the targeted purification of pharmaceutically active compounds from plant sources [21,22,23,24,25,26]. A single peak of the compound in the HPLC analyses of a retention time of 9.052 min showed the desired activity (Figure 3B). The purified compound is evidently a novel compound that has cytotoxic activity. The IC_50_ value of the purified bioactive compound (oroxyquinone) was determined, and was found to be 58.9 µM (95% CI = 54.5 to 63.7 µM), as shown in Figure 3C,D. The structure was elucidated based on spectroscopic techniques. The chemical characterization of the purified bioactive compound using NMR, FT-IR, and MS revealed that the purified phytocompound is 2-[p-(2-Carboxyhydrazino)phenoxy]-6-(hydroxymethyl) tetrahy-dro-2H-pyran-3,4,5-triol, with a chemical formula of C13H18N2O8 (exact molecular mass = 330.11), and the chemical structure is as shown in Figure 4. The searching of 2-[p-(2-Carboxyhydrazino)phenoxy]-6-(hydroxymethyl) tetrahy-dro-2H-pyran-3,4,5-triol using chemical databases—namely SciFinder, ChemSpider, Pubchem, and Pubmed—revealed that the purified phytocompound from OI is a novel compound. Therefore, the purified bioactive compound was named (common name) “Oroxyquinone” based on the source of purification (*Oroxylum indicum*) and the chemical structure (glycosylated hydroquinone) for convenience. The overall finding that oroxyquinone induces gDNA fragmentation, DNA condensation, the arrest of the cell cycle at the S phase, and an increase in the annexin-V positive cells in dose-dependent manner clearly suggests that the novel oroxyquinone induces apoptosis in PC3 cells.

In view of the fact that the majority of these cancer-related deaths are due to tumor metastasis rather than the primary tumors [28], we also investigated the anti-metastatic activity of oroxyquinone on PC3 cells which are highly metastatic in vitro, using a wound-healing assay. The results showed that oroxyquinone significantly inhibits the highly metastatic PC3 cells’ migration by approximately ≈35% at 6 h and ≈55% at 12 h. These findings suggest that oroxyquinone can be developed as a therapeutic drug for the management of both primary tumour growth and metastasis.

The absence of caspase-3 and PARP cleavage indicated an apoptotic mechanism independent of the two proteins. As discussed by Galluzii et al., 2012, there are various mechanisms of cell death, and caspase-independent cell death is one of the mechanisms [2]. AIF can be localized from the mitochondrial membrane to the nucleus following cellular stresses, and it induces gDNA fragmentation, which correlates with our observation of AIF being enriched in the nuclear fraction following treatment with oroxyquinone (Figure 9). The results clearly indicate that oroxyquinone induces apoptosis in PC3 cells in a caspase-independent manner. Most cancer cells have a tendency to evade the classical process of apoptosis [29,30,31], and targeting alternate pathways to induce apoptosis to cancerous cells is a more recent development [32,33].

Our finding of the non-involvement of caspase and PARP in apoptosis is in accordance with the studies carried out by various researchers [34,35,36,37] using phytochemicals as well as amino acids, which suggests that PC3 cells have the potential to undergo both caspase-dependent and -independent pathways in apoptosis.

Furthermore, the involvement of Mitogen-Activated Protein Kinases (MAPK) pathways was assessed by Western blotting. MAPKs respond to a wide range of extracellular and intracellular changes. The stimulation of JNK and p38 trigger both antiapoptotic and pro-apoptotic responses, depending on the type of cell and the stimulator [38]. It was originally shown that ERKs are important for cell survival, whereas JNKs and/or p38 were deemed stress-responsive and involved in apoptosis. However, the regulation of apoptosis by MAPKs is more complex than initially thought, and is often controversial [39]. It has been reported that JNK activation triggers apoptosis in response to many types of stress, including UV and radiation, protein synthesis inhibitors, and anticancer drugs (cisplatin, adriamycin or etoposide). In most cases, p38 is simultaneously activated with JNKs [40]. The stimulation of p38 and JNK signaling pathways by oroxyquinone in PC3 cells in a dose-dependent manner suggests that cells require p38 and/or JNK activations for apoptosis induction. ERK is abruptly stimulated by oroxyquinone at a high dose (120 µM) but not at low doses (30 µM and 60 µM), even though apoptosis is significantly induced at low doses (Figure 10A). Therefore, it is likely that the ERK stimulation at the highest dose of oroxyquinone is not responsible for the apoptosis induction in PC3 cells. MTT assays were conducted in the presence and absence of specific inhibitors of p38, JNK, and ERK to confirm the functional roles of p38, JNK, and ERK involved in the oroxyquinone induced apoptosis in PC3 cells. The results revealed that the presence of the specific inhibitor of p38 (SB203580) abrogates the oroxyquinone-induced inhibition of PC3 cell proliferation, whereas the presence of specific inhibitors for JNK (SP600125) or ERK (U0126) enhances the inhibition of PC3 cells’ proliferation (Figure 10B). The dependence of p38 in oroxyquinone-induced apoptosis is further confirmed by the use of SB202190, where a statistically significant recovery from cell death was observed (Figure 10C). Taken together, the findings strongly suggest that oroxyquinone induces apoptosis through the p38 pathway and cell cycle arrest in prostate cancer cells. The activation of p38 in caspase-independent cell death was observed earlier in various studies [41,42,43,44], and in an AIF dependent manner [45]. Our findings evidence the involvement of AIF and the independence of caspase and PARP in cell death induced by the novel compound. The activation of JNK by oroxyquinone may have other cellular effects in PC3 cells. However, a detailed investigation of the mechanism of the novel compound is warranted.

## 4. Materials and Methods

### 4.1. Cell Culture

PC3 cells are androgen-independent metastatic adenocarcinoma cells isolated from bone metastases. The cells were obtained from the National Centre for Cell Science (NCCS) in Pune, India. The cells were cultured in RPMI 1640 (GIBCO, LifeTechnologies, Waltham, MA, USA) supplemented with 10% FBS (GIBCO, LifeTechnologies) and 1U PenStrep (GIBCO, LifeTechnologies) in cell culture plates. The cells were maintained in a humidified chamber with 95% atmospheric air and 5% CO_2_ at 37 °C.

### 4.2. Extraction and Purification

The plant specimen was collected from Khurukhul, Manipur, India; was identified by the Botanical Survey of India, Eastern Regional Centre, Ministry of Environment and Forests, Shillong, India; and a voucher specimen was deposited under Voucher No. BSI/ERC/Tech/2016/463. The leaf of OI was collected, dried under shade, and ground using a blender. Gross particles like midribs were removed before processing. In total, 25 g of the leaf powder was weighed and subjected to aqueous extraction using a soxhlet apparatus (500 mL capacity) below 60 °C for the preliminary investigation of the inhibition of cell proliferation. For the purification of the phytocompound, 500 g of the leaf extract was weighed and subjected to hexane followed by chloroform extraction using a 20,000 mL capacity soxhlet apparatus. The crude extract was then condensed using a rotary vacuum evaporator, followed by freeze-drying using a lyophilizer. The chloroform extract was subjected to fractionation using water at a final concentration of 50:50 Chl:H_2_O, and the fractions were lyophilized. The lyophilized powders were used for bioassay for testing cells antiproliferative activity or stored at −20 °C until they were used. The antiproliferative assays were tested on cultured cells using an MTT assay, as described below. A concentration of 100 mg/mL of the aqueous fraction was prepared in water for further purification using silica gel chromatography. The initial fractionation was conducted on a silica column chromatograph (230–400 mesh, 40 × 600 mm). In total, 5 mL of 100 mg/mL crude extract was loaded in the column, and fractions of 50 mL were collected using water as the mobile phase. Each fraction was tested for cytotoxicity on cultured cells. The fraction showing bioactivity was further purified using HPLC (Agilent 1260 Infinity) on a Zorbax C-18 column of 4.5 × 150 mm with a 5 µm particle size. Flow rate of 1 mL per minute was maintained with 8% methanol 92% water in an isocratic mode for 10 min, followed by a gradual increase in the percentage of methanol to 100% in 40 min. Each peak was collected in the time slice mode for 1 mL each, and was analyzed for cytotoxic activity. The peaks which showed significant bioactivity on cultured cells were collected and used for further assays.

### 4.3. Cell Viability Assay (MTT Assay)

Approximately 1 × 10^3^ cells were seeded in a 96-well tissue culture plate. The cells were cultured in 100 µL RPMI (without phenol red) containing 10% FBS, at 5% CO_2_, and and incubated at 37 °C. The cells were treated for 24, 48 and 72 h with 10 µL of solution containing various doses of crude or purified compound in triplicates. The experiment was also conducted in the presence or absence of specific inhibitors. The control cells were treated with 10 µL PBS under identical conditions to the treated cells. At the end of treatment, 10 μL 3-(4,5-dimethylthiazol-2-yl)-2,5-diphenyltetrazolium bromide (MTT, 5 mg/mL) was added to each well and incubated at 37 °C for 4 h. The MTT crystals formed were dissolved in 100 µL SDS-HCl solution and incubated for 4 h at 37 °C. The number of viable cells were then quantified by measuring the absorbance at 570 nm on a microplate reader (Multiskan Go, Thermo Fisher Scientific, Waltham, MA, USA). The experiment was conducted three times in triplicate.

### 4.4. gDNA Fragmentation Assay

gDNA fragmentation assays were carried out as previously described [46]. Approximately 5 × 10^6^ PC3 cells were seeded in 6-well tissue culture plates and treated with 50, 200, and 400 µg/mL leaf crude extract of OI. The control cells were treated with PBS as the negative control. Following the treatment, the cells were lysed using lysis buffer and subjected to electrophoresis on 2% agarose gel. The images were recorded on an imager (ChemiDoc, BioRad, Hercules, CA, USA).

### 4.5. Chemical Characterization

The bioassay-guided purified compound was analyzed using an Agilent 6200 LC/MS system. The purified compound was subjected to LC using 5% methanol and 95% water isocratic flow with proshell 120, a C-18 column of 4.6 × 50 mm dimensions, and a 2.7 µm particle size maintained at 30 °C, with a flowrate of 1 mL per minute. The desired peak was selected and nebulized at 300 °C, 40 psig, analyzed at 135 v for a mass range between 100–1500 *m*/*z*.

All of the NMR spectra were recorded on Bruker-Avance 500 MHz spectrometer with TMS as an internal standard. The 1H chemical shifts are reported in delta (δ) units, in parts per million (ppm) downfield from tetramethylsilane, and also with reference to the residual protic solvent (CDCl3, δH = 7.26 ppm). The 13C chemical shifts are referenced to the solvent signal (CDCl3). Infrared (IR) data were recorded as films on potassium bromide (KBr) pellets on a FT-IR spectrometer. The absorbance frequencies are reported in reciprocal centimeters (cm^−1^). The high-resolution mass spectra (HRMS) were performed on a VG Autospec-3000 spectrometer. The chemical structure and IUPAC name of the purified phytocompound were elucidated using ChemDoodle Software (iChemLabs, Ver. 9.0.3). In order to test whether the purified phytocompound is a new chemical compound, chemical databases—namely SciFinder, ChemSpider, Pubchem, and Pubmed—were used.

### 4.6. Comet Assay

The comet assay was conducted as per the protocol described by Olive and Banath, 2006 [47]. Briefly, the cells were treated with various doses of HPLC-purified compounds or PBS as a negative control for 24 h. The cells were trypsinized, and approximately 5 × 10^3^ cells were suspended in PBS, mixed with 1.2 mL low-melting agarose at 40 °C by gentle pipetting, and poured on an agarose pre-coated microscope slide. After allowing the solidification of the agarose at room temperature, the slide was submerged in neutral lysis solution (2% SDS, 0.5 M EDTA, and 0.5 mg/mL proteinase K) and incubated at 37 °C for 16 h in the dark. The slides were then washed three times with neutral electrophoresis buffer, followed by electrophoresis at 0.6 V/cm for 25 min. The slides were then stained with propidium iodide (50 µg/mL) and observed under a fluorescent microscope (Leica Microsystem GmbH, Wetzlar, Germany), and the images were recorded. The tail lengths of at least 25 comets in each slide were scored using Leica Application Suite (LAS), Leica Microsystem, GmBH, Germany software for analysis.

### 4.7. Annexin-V Analysis

PC3 cells were cultured as described earlier and harvested after treating the cells with 30, 60 and 120 µM of oroxyquinone. The harvested cells were washed once with PBS, followed by binding buffer (10 mM HEPES, pH 7.4, 140 mM NaCl and 2.5 mM CaCl_2_). The cells were suspended at a count of 1–5 × 10^6^ cells/mL. 5 µL of Annexin V antibody (Invitrogen Cat No. 88-8005-72) was added to 100 µL of the cell suspension and incubated for 10 min in the dark. A further 2 mL of binding buffer was added to each tube and centrifuged at 400× *g* for 5 min in room temperature. The pellets were further resuspended in 200 µL 1X binding buffer and 5 µL of 500 µg/mL PI was added before analysis with a flow cytometer.

### 4.8. Wound Healing Assay

The wound healing assay was conducted as described previously by Rodriguez and colleagues [48]. The cells were seeded in 6-well tissue culture plates and grown overnight; the bottom surface was scratched with a sterile pipette tip, washed with PBS, and treated with 5 µg/mL of the purified phytocompound. After the treatments, the cells were observed in a microscope, and pictures were taken at 0, 6, and 12 h.

### 4.9. Cellular Fractionation

PC3 cells cultured in a 60-mm cell culture dish were harvested by trypsinization and resuspended in fractionation buffer containing 20 mM HEPES (pH 7.4), 10 mM KCl, 2 mM MgCl2, 1 mM EDTA and 1 mM EGTA. The cells were lysed by passing them through a 27-guage needle 10 times. The nuclear-enriched pellet fraction was collected by centrifuging the lysate at 720× *g* for 5 min; the supernatant contained mitochondrial, cytoplasmic and membrane fractions. The nuclear pellet was washed with 500 µL fractionation buffer and passed through a 25-gauge needle 10 times, followed by centrifugation at 720× *g* for 10 min. The pellet was collected and suspended in TBS with 0.1% SDS, and homogenized. The supernatant containing cytoplasmic, mitochondrial and membrane components was centrifuged at 10,000× *g* for 5 min, and the pellet containing the mitochondrial fraction was collected and lysed in TBS with 0.1% SDS.

### 4.10. Western Blots

Cells were seeded on 6-well tissue culture plates and treated with the purified compound or PBS as a control for 24 h. The cells were trypsinized and lysed, and an equal amount of total proteins were separated by 10% SDS-PAGE. After transferring the separated proteins onto the membrane, they were probed with anti-PARP, anti-caspase 3, AIF, anti-ERK, anti-P38, and anti-JNK antibodies. All of the antibodies used in the Western blots were obtained from Cell Signalling, Danvers, MA, USA. The fluorescence signals were exposed to a photo film or directly recorded on an imager (Chemidoc, BioRad).

### 4.11. Cell CYCLE Analysis

The cells were seeded in 6-well tissue culture plates and treated with 20, 40, and 80 µg/mL of the purified plant compound for 16 h. After treatment, the cells were harvested by trypsinization, washed with PBS, and fixed with 70% ethanol for 30 min at 4 °C. The cells were washed with PBS, treated with 100 µg/mL RNase, stained with propidium iodide (50 µg/mL), and then the cell cycle was analyzed on a flow cytometer (BD Acuri C5).

### 4.12. DAPI Staining

PC3 cells were cultured on sterile polylysine-treated slide covers in RPMI supplemented with 10% FBS at 37 °C for 16 h after treatment with various doses of oroxyquinone. The cells were washed with PBS to remove residual media, stained with 300 nM of DAPI, and incubated in the dark for 5 min before observation under a fluorescent microscope.

### 4.13. Statistical Analysis

The data obtained in the above studies were statistically analyzed and expressed as a mean ± SD. The statistical significance was assessed by ANOVA, followed by Tukey–Kramer multiple comparison tests using GraphPad Prism 8.0. The significance level was set at *p* < 0.05. The treated groups were compared with the untreated controls in the MTT assay and comet assay. In the wound healing assay, the treated groups were compared with the untreated controls at the same time points.

## 5. Conclusions

Regarding cancer, being a frontier area of research, there has been significant development in the screening and discovery of potential anticancer drugs. We have purified a novel cytotoxic phytocompound, 2-[p-(2-Carboxyhydrazino)phenoxy]-6-(hydroxymethyl) tetrahydro-2H-pyran-3,4,5-triol that induces apoptosis in PC3 cells. The activation of MAPK pathways is required for apoptosis induced by oroxyquinone. In addition to the cytotoxic activity, oroxyquinone also has an anti-metastatic activity. Regarding India, being a large nation with considerable potential in the development of nutraceuticals, the findings of the current study will benefit the improvement of medicinal plants for use as therapeutic agents.

## Data Availability

Data is contained within the article.

## Supplementary Materials

The following are available online at https://www.mdpi.com/article/10.3390/ph15050559/s1, Figure S1: DNA fragmentation of PC3 cells treated with a crude extract of OI. Figure S2: FT-IR chromatogram of oroxyquinone. Figure S3: NMR chromatogram of oroxyquinone. Figure S4: Western blot image of Caspase-3 and PARP after treatment with Oroxyquinone.
