# Supplementary material for: Bioassay-Guided Isolation of 2-[p-(2-Carboxyhydrazino)phenoxy]-6-(hydroxymethyl)tetrahydro-2H-pyran-3,4,5-triol from Oroxylum indicum and the Investigation of Its Molecular Mechanism Action of Apoptosis Induction
Source: Pharmaceuticals (Basel). 2022 Apr 30;15(5):559. doi: 10.3390/ph15050559 (PMC9148098; doi:10.3390/ph15050559)
Supplement: Supplementary file 1 [file pharmaceuticals-15-00559-s001.zip › pharmaceuticals-1487154-supplementary.pdf]

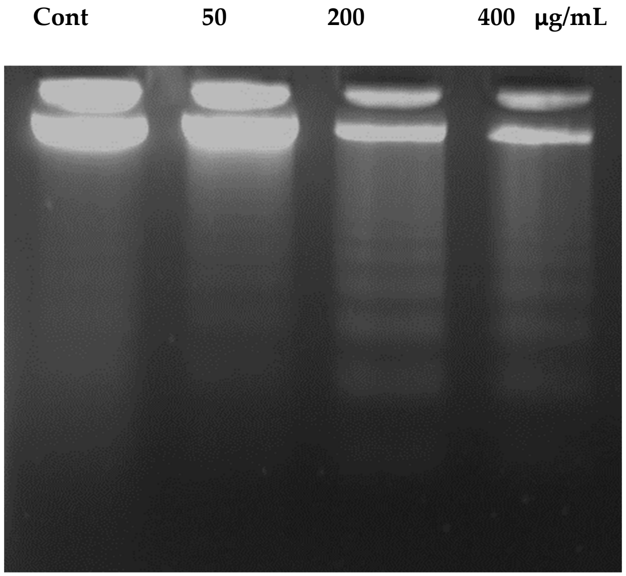


**Figure S1.** The leaf crude extract induced gDNA fragmentation in PC3 cells at 24 h of treatment in a dose-dependent manner.

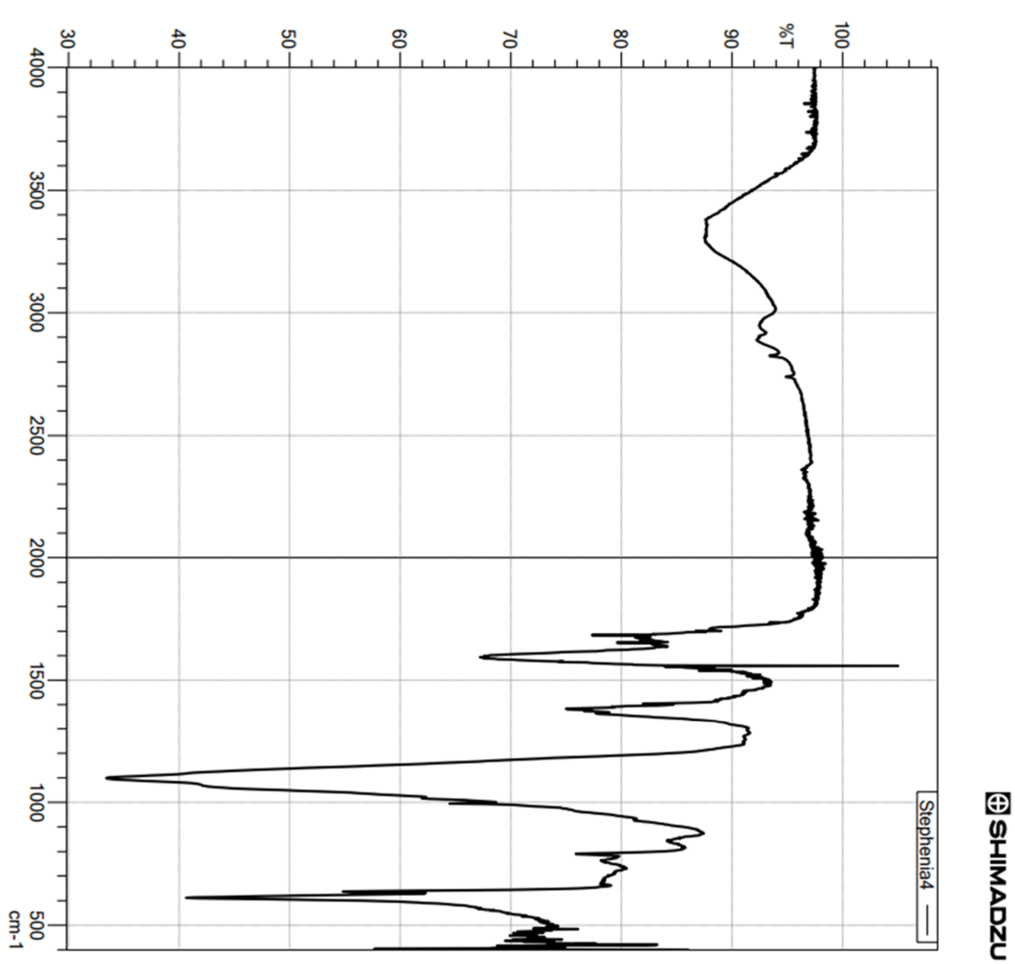


C:\Program Files\LabSolutions\IR\Data\Stephenia4.ispd

| | Item | Value |
|---|---|---|
| 2 | Sample name | met |
| 3 | Sample ID | |
| 4 | Option | |
| 5 | Intensity Mode | %Transmitance |
| 6 | Apodization | None |
| 9 | No. of Scans | 80 |

1

**Figure S2.** FTIR Spectra.

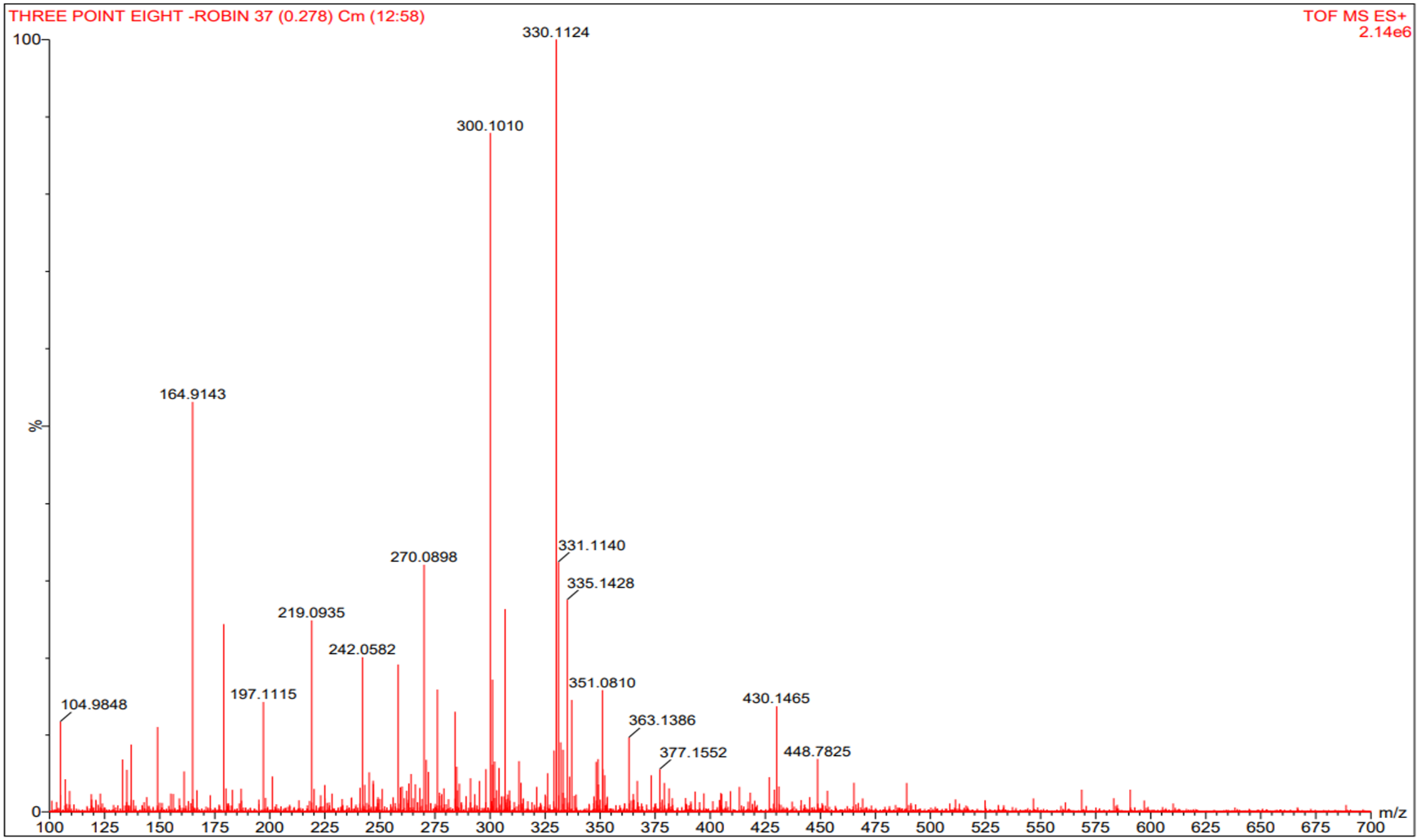


**Figure S3.** NMR spectra.

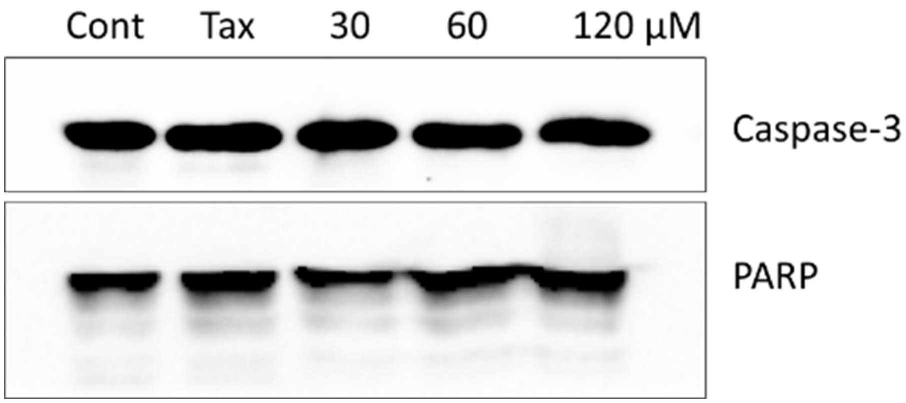


**Figure S4.** Oroxyquinone induces caspase-3 and PARP independent apoptosis in PC3 cells: PC3 cells were cultured and treated with different doses of oroxyquinone, total proteins extracted and separated by SDS-PAGE and immunoblotted using antibodies against procaspase-3 and PARP.
